# Brain-age estimation with a low-cost EEG-headset: effectiveness and implications for large-scale screening and brain optimization

**DOI:** 10.3389/fnrgo.2024.1340732

**Published:** 2024-04-24

**Authors:** John Kounios, Jessica I. Fleck, Fengqing Zhang, Yongtaek Oh

**Affiliations:** ^1^Department of Psychological and Brain Sciences, Drexel University, Philadelphia, PA, United States; ^2^Department of Psychology, Stockton University, Galloway, NJ, United States

**Keywords:** brain-age estimation, brain health, EEG, machine learning, resting-state EEG

## Abstract

Over time, pathological, genetic, environmental, and lifestyle factors can age the brain and diminish its functional capabilities. While these factors can lead to disorders that can be diagnosed and treated once they become symptomatic, often treatment is difficult or ineffective by the time significant overt symptoms appear. One approach to this problem is to develop a method for assessing general age-related brain health and function that can be implemented widely and inexpensively. To this end, we trained a machine-learning algorithm on resting-state EEG (RS-EEG) recordings obtained from healthy individuals as the core of a brain-age estimation technique that takes an individual's RS-EEG recorded with the low-cost, user-friendly EMOTIV EPOC X headset and returns that person's estimated brain age. We tested the current version of our machine-learning model against an independent test-set of healthy participants and obtained a correlation coefficient of 0.582 between the chronological and estimated brain ages (*r* = 0.963 after statistical bias-correction). The test-retest correlation was 0.750 (0.939 after bias-correction) over a period of 1 week. Given these strong results and the ease and low cost of implementation, this technique has the potential for widespread adoption in the clinic, workplace, and home as a method for assessing general brain health and function and for testing the impact of interventions over time.

## 1 Introduction

This report describes an electroencephalogram-based machine-learning technology for assessing whether an individual's brain is aging more quickly or more slowly than would be expected relative to healthy individuals of the same age. Our method provides a window on to the combined effects of physiological, pathological, genetic, environmental, and lifestyle factors on brain aging. Because we implemented this method with a low-cost, user-friendly electroencephalogram (EEG) headset, it can be used in the workplace, clinic, or at home to track an individual's general neurological health and functioning over time to assess the impact of factors that can potentially affect brain health and aging.

Measuring and understanding how brain age is important for identifying individuals at risk for disease or decline and flagging them for examinations to detect and diagnose disorders that are more difficult to treat if detected later during disease progression. It is also important for understanding how neurological disorders, injuries, and environmental insults may prematurely age a brain and how particular lifestyles may preserve or enhance it (Cole et al., 2019). Research has revealed several manifestations of brain aging, including mitochondrial dysfunction; dysregulated energy metabolism; impaired DNA repair; inflammation; physical trauma; infection; and toxins (Mattson and Arumugam, 2018). In particular, magnetic resonance imaging (MRI) studies have identified structural biomarkers of aging such as demyelination of axons and decreases in gray-matter and white-matter volumes that can contribute toward the estimation of a brain-age index of general neurological health and function (Cole et al., 2017). As shown below, a few minutes of resting-state EEG (RS-EEG) recorded with a low-cost headset can also yield an accurate estimation of a person's brain age.

When you meet someone for the first time, you might try to estimate their age. You can do this by looking at particular features of their appearance: Is their hair gray? Do they have wrinkles? When you learn their actual age, you might be surprised. Based on their appearance, you might judge that they are aging more quickly or more slowly than would be expected.

Machine-learning (ML) algorithms can do this, too. For example, ML algorithms have been trained on large datasets of features extracted from MRI brain images to identify patterns of features that enable classification of individual cases (Cole et al., 2017). The approach is to input a large number of healthy individual cases (i.e., the *training set*) along with the chronological age of each subject. This enables the algorithm to learn which combinations of MRI features predict chronological age and which do not. The resulting statistical model is then used to predict the chronological age of other individuals (i.e., the *test set*), healthy or otherwise, based on features of their MRIs.

One important outcome is that for some individuals, predicted brain age is older than chronological age while for others, predicted brain age is younger than chronological age. These individuals are said to have a *brain-age gap* (BAG), which is the estimated brain age minus the chronological age.

Differences between chronological and estimated brain ages can be partly due to statistical noise in the brain-age predictions; the relationship between chronological age and the neuroimaging features and modeling techniques used to estimate brain age includes statistical noise and is therefore not perfectly accurate (see de Lange et al., 2022). But statistical noise does not completely explain brain-age gaps because BAGs correlate with independent measures of cognitive function and risk for dementia onset (e.g., Kaufmann et al., 2019; Biondo et al., 2022) and because some diseases, such as schizophrenia and dementia, are known to be associated with larger BAGs in affected persons (Cole et al., 2017; Bijsterbosch, 2019; Hou et al., 2019). Thus, an important goal for any ML model of brain aging is to minimize noise, for example, by improving feature selection, sample age range, and so forth (de Lange et al., 2022) so that a deviation between a person's chronological age and predicted brain-age can be attributed to slower-than-average or quicker-than-average aging rather than to noise.

Targeted treatment for a disease typically depends on a specific diagnosis. Unfortunately, precise diagnoses of neurological disorders of aging such as Alzheimer's disease are complicated by the fact that the symptomatology of a disease can vary across individuals and that more than one disease is often present in the same patient (Coulthard and Love, 2018). Additionally, significant physical change can occur in the brain before clinical symptoms are manifested by the affected person (Sperling et al., 2014). Thus, a global measure of age-related brain health—analogous to the idea of IQ as a global measure of cognitive ability relative to peers—that reflects contributions from multiple diseases, injuries, etc. can be used to screen individuals at an early phase of brain deterioration when interventions may be more effective. Additionally, healthy individuals may repeatedly estimate their brain age after implementing lifestyle interventions such as dietary changes, exercise regimens, types of meditation, sleep adjustments, and cognitive training programs to assess whether any single intervention or combination of interventions can slow or reverse brain aging. This may be particularly helpful for people interested in optimizing performance, such as athletes, executives, and students.

Due to technical refinements, brain-age estimates based on structural MRI continue to improve (Wood et al., 2022). Nevertheless, there are two limitations to the practical use of structural MRIs to predict brain age. One limitation is that biomarkers of structural brain aging do not always correlate well with cognitive function. This is exemplified by the poorly understood phenomena of cognitive reserve and neural reserve whereby a brain that exhibits Alzheimer's-related amyloid pathology and structural damage can retain a high level of function (Bennett, 2017; Fleck et al., 2017). Thus, it is not always clear which structural changes in the brain may impair neural function in ways that adversely affect cognitive function. For example, a scratch on a laptop computer is unlikely to affect a computer's performance. Moreover, cognitive function is more important for unencumbered living than is a structurally pristine brain. This means that estimates of structural brain-age, though useful and informative, can be an incomplete method for assessing or predicting decline of brain function.

Another limitation is cost. Although there are currently limited options for the treatment of age-related neurological disorders such as Alzheimer's disease, current and future interventions may be more effective if nascent neurological disorders could be detected very early—even presymptomatically—while they may be more treatable. It would also be helpful to be able to assess brain health at regular intervals to confirm healthy aging, to detect a deviation in the trajectory of aging, or to monitor changes following diagnosis or intervention. In these scenarios, MRI will not be practical because widespread early and repeated screening would be very expensive and unlikely to be covered by insurance companies.

Functional brain-age estimation via EEG is an attractive alternative to structural brain-age estimation with MRI. EEG measures the synchronous activity of large populations of neurons via electrodes placed on the scalp (Biasiucci et al., 2019). Instead of imaging brain structure, EEG images brain function, and has been shown to be effective in capturing the contribution of cognitive reserve to brain health and cognitive function (e.g., Bozzali et al., 2015; Varela-López et al., 2022). Furthermore, EEG hardware is now inexpensive and user-friendly enough that its adoption is economically practical for the purpose of widespread screening and use in the workplace or at home. For this reason, and because of the high cost of MRI, some research groups have begun to direct their efforts toward brain-age assessment using EEG (e.g., Dimitriadis and Salis, 2017; Al Zoubi et al., 2018).

These initial efforts have shown encouraging, but mixed, results. For example, using archival EEG datasets, Dimitriadis and Salis (2017) obtained good brain-age prediction accuracy within those datasets (*R*^2^ = 0.60 for eyes-open, *R*^2^ = 0.48 for eyes-closed), though they did not provide satisfactory external validation of their training-set model by applying their model to a separate test-set of sufficient size. Furthermore, there was no documentation of the health status of the participants in one of these training sets to ensure that they were all healthy. Al Zoubi et al. (2018) used a large dataset of EEGs obtained from participants during MRI scanning. Their mean absolute error (MAE), a measure of prediction accuracy, was 6.87 years. However, the practical usefulness of their findings is limited by the fact that their subjects were reclining in a noisy and confining MRI scanner during the EEG recordings which may limit the generalizability of their results to participants outside of a scanner. Sun et al. (2019) used large training- and test-sets of EEGs obtained while participants slept and obtained brain-age estimates that had a MAE of 8.1 years. Engemann et al. (2022) compared various machine-learning and deep-learning methods for brain-age estimation based on a large, combined set of archival EEG recordings (53 EEG channels per recording) and obtained best cross-validated MAE values from 7–8 years and *R*^2^ values up to ~0.75. Most recently, Banville et al. (2023) implemented ML-based brain-age prediction using EEG data collected with a low-cost headset during meditation and sleep and achieved a cross-validated *R*^2^ between 0.3 and 0.5, although with low test-retest reliability over relatively short intervals (i.e., days).

Our goal was to develop an accurate ML model to predict individual participants' brain ages based on data recorded with a low-cost EEG headset, the EMOTIV EPOC X (referenced as the headset moving forward; EMOTIV Inc., San Francisco, CA). We recorded resting-state EEGs (RS-EEGs) from participants using the low-cost, mobile wireless EEG headset; a subset of these recordings was used in the training set and the remainder in the test set (detailed below). These EEGs were recorded from healthy participants who were at “rest,” that is, awake but without a task to perform. Additional recordings from healthy participants completed in several different laboratories using a variety of recording systems were included in the training set. RS-EEGs are known to reflect stable, trait-like individual differences in cognition, personality, and psychopathology (John et al., 1988; Erickson et al., 2018). We extracted a variety of features from these EEGs and subjected them to several types of ML algorithms plus statistical bias-correction. The machine-learning model that we derived from the training set was then applied to our independent test set.

## 2 Methods

### 2.1 Stockton Bryn Mawr Emotiv dataset

We collected the Stockton Bryn Mawr Emotiv (SBE) dataset for this project. This dataset includes recordings from 91 healthy participants, collected at three locations: Stockton University (New Jersey), Bryn Mawr College (Pennsylvania), and online via EmotivLABS. A description of the sample from each testing location is provided in Table 1. Stockton University participants were recruited using a social media advertisement. Bryn Mawr College participants were recruited using an older adult participant database. Online participants were recruited via EmotivLABS, Emotiv's online platform for data collection. Registered users on this platform passed a certification test that ensures they could collect sufficient quality data (Williams et al., 2023).

**Table 1:** SBE dataset demographics.

	**Stockton University *n* = 35**	**Bryn Mawr College *n* = 20**	**Emotiv LABS *n* = 36**
**Age**			
Range	30–64 years	32–85 years	30–63 years
Mean (SD)	45.17 (10.67)	71.10 (13.31)	39.28 (8.49)
**Years of education**			
Mean (SD)	16.63 (1.75)	16.80 (2.78)	16.67 (2.24)
**Gender**			
Female	29	15	17
Male	6	5	18
Other	–	–	1
**Race/Ethnicity**			
Caucasian	30	20	23
Hispanic	4	–	3
Asian	1	–	10
**Handedness**			
Right	35	19	26
Left	–	1	7
Ambidextrous	–	–	3
**Employment**			
Employed	33	5	36
Unemployed	2	–	–
Retired	–	15	–

### 2.2 Supplemental training datasets

In addition to the subset of our SBE dataset used in the training set, ML model training was based on four previously recorded EEG datasets, totaling 505 participants. Two of the datasets (Stockton A and B) were recorded in the second author's lab and two are publicly available. All participants gave written informed consent. Two sessions of data were available for 91 participants, and both session 1 and session 2 recordings for these participants were included as separate points in the training set for a total of 596 recordings. Gender and handedness were noted but not used as features for training or testing the machine-learning model. A brief description of each dataset is provided below and summarized in Table 2.

**Table 2:** Supplemental training datasets.

**Dataset**	***N***	**Age**	**Gender**	**Handedness**
**Stockton A**				
Session 1	163	46–93 years	Female: 109	Right: 163
		63.57 (8.61)	Male: 54	
Session 2	57	55–85 years	Female: 37	Right: 57
		68.86 (8.08)	Male: 20	
**Stockton B**				
	123	35–83 years	Female: 89	Right: 109
		56.64 (7.53)	Male: 34	Left: 14
**CHBM**				
	143	30–68 years	Female: 56	Right: 122
		37.87 (7.32)	Male: 87	Left: 13
				Mixed: 5
				Unavailable: 2
**SRM**				
Session 1	76	30–71 years	Female: 45	Unavailable
		44.43 (11.36)	Male: 31	
Session 2	34	30–65 years	Female: 21	Unavailable
		45.54 (9.76)	Male: 13	
**Total**				
	596	30–93 years	Female: 357	Right: 451
		53.03 (14.06)	Male: 239	Left: 27
				Mixed: 5
				Unavailable: 113

#### 2.2.1 Stockton A

This dataset contains EEG recordings from 163 healthy adults, ages 46–93 years, recorded at Stockton University; 57 subjects participated in a second recording session ~1 year after the first session. Both sessions used a 128-channel sensor net with a Cz reference. Session 1 data were recorded with an EGI Net Amps 300 amplifier (Electrical Geodesics, Inc.) with a 250-Hz sampling rate; session 2 data were recorded with an EGI Net Amps 400 amplifier (Electrical Geodesics, Inc.) and 500-Hz sampling rate. Each recording included 3 min of eyes-open and 3 min of eyes-closed resting-state EEG.

#### 2.2.2 Stockton B

This dataset contains EEG recordings from 123 healthy adults, ages 35–83 years, recorded at an urban satellite campus of Stockton University. The EEG data were recorded using a 128-channel Geodesic Sensor Net with Cz reference. The data were recorded with an EGI Net Amps 400 amplifier (Electrical Geodesics, Inc.) and 500-Hz sampling rate. Each recording included 3 min of eyes-open and 3 min of eyes-closed resting state data.

#### 2.2.3 Cuban human brain mapping project

This dataset contains EEG recordings from 282 healthy adults, ages 18–68 years, recorded in La Habana, Cuba (Valdes-Sosa et al., 2021). The recording task included baseline (eyes-open and eyes-closed resting state), reactivity, hyperventilation, and recovery states. We selected recordings from participants 30 years of age or older for our training set (*N* = 143). Recordings were made using 64 or 120 electrodes with a linked-earlobes reference. EEG data were recorded using a MEDICID 5 system with a 200-Hz sampling rate. The data were filtered with 0.5–50 Hz band-pass and 60-Hz notch filters. Each recording included 5 min of eyes-open and 15 min of eyes-closed data.

#### 2.2.4 Stimulus-selective response modulation project

This dataset contains resting-state EEG recordings from 111 healthy adults, ages 17–71 years, collected in conjunction with the Stimulus-Selective Response Modulation (SRM) Project at the University of Oslo, Norway (Hatlestad-Hall et al., 2022). A subset of the subjects participated in a second recording session 2–3 months after the first session. We selected participants who were 30 years of age or older (*N* = 76); 34 of these participants completed a second recording session. EEGs were recorded with 64 electrodes placed according to the International 10–10 System. The EEG data were recorded using the BioSemi ActiveTwo system (Biosemi B.V., Amsterdam), with the system's zero-reference, and a 1,024-Hz sampling rate. The data were re-referenced to average reference. Each recording included 4 min of eyes-closed resting-state data.

### 2.3 Materials

**Figure 1:**
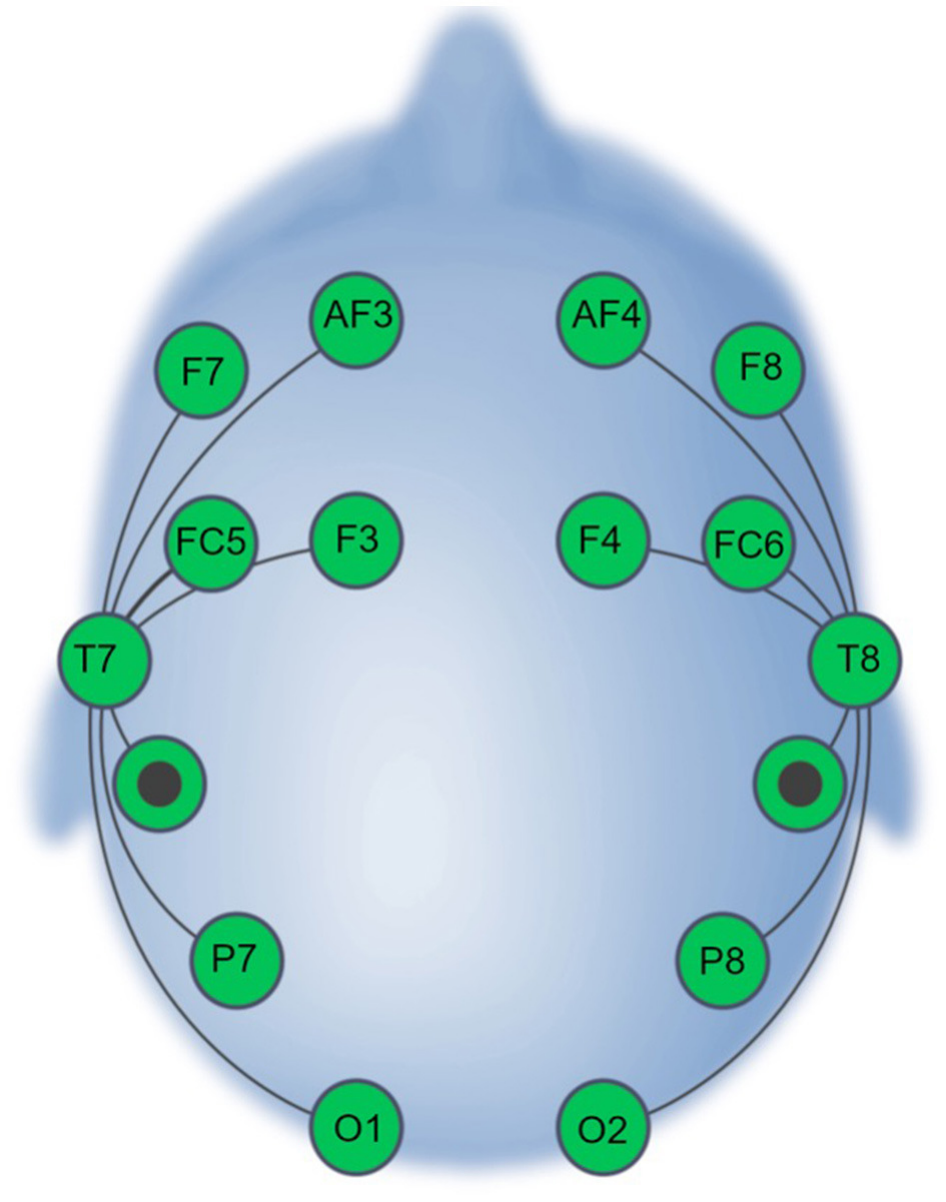
EPOC X Electrode Montage (adapted from Williams et al., 2023).

Session 1 and Session 2 scripts were prepared and deployed using the EmotivPRO v. 3.0 (https://emotiv.gitbook.io/emotivpro-v3/) software. The EPOC X headset (https://emotiv.gitbook.io/epoc-x-user-manual/) was used for all recordings in the SBE dataset. The headset uses 14 saline-soaked felt sensors (electrode sites AF3/4, F3/4, F7/8, FC5/6, T7/8, P7/8, and O1/2) and reference sensors at P3/P4 (Figure 1). The data were recorded with a 0.2–45.0 Hz bandpass filter and either a 50-Hz or a 60-Hz notch filter (depending on the country in which the data were recorded).

### 2.4 Recording procedure

The data collection procedure was approved by the Bryn Mawr College, Stockton University, and Drexel University Institutional Review Boards. Participants completed 2 sessions, scheduled ~1 week apart. The EEG recording procedure was the same for sessions 1 and 2.

After providing informed consent, participants completed demographics and handedness questionnaires. Next, the headset was positioned, and contact quality (i.e., impedance) and EEG quality (a combination of contact quality, signal quality over time, and signal magnitude quality) checks were performed (see EmotivPRO user manual). The EEG data were recorded at Stockton University and Bryn Mawr College via a USB receiver dongle with 16-bits and 128-Hz resolution and a 60-Hz notch filter.

All instructions were presented on the computer screen and read aloud by the experimenter. Participants completed three identical blocks of resting-state EEG recordings. Recordings alternated between eyes-open and eyes-closed in 1-min intervals for a total of two eyes-open and two eyes-closed recordings per block. Each block concluded with an arousal rating during which participants were asked, “On a scale of 1 to 5, 1 being very tired and struggling to stay awake and 5 being very alert, how would you rate your arousal level at the end of this block?” There was a brief break between blocks to reduce drowsiness. Across blocks, participants completed 12 min of resting-state EEG, 6 min with eyes open and 6 min with eyes closed. Participants were compensated $50 per session.

All procedures involving human participants adhered to the ethical standards delineated by the institutional and/or national research committee and in compliance with the 1964 Helsinki declaration and its later amendments or comparable ethical standards. Informed consent was obtained from all participants.

### 2.5 Online data collection procedure

The experiment protocols for sessions 1 and 2 were published on EmotivLABS on the Citizen Science page (see https://labs.emotiv.com/) and were visible between June 15 and August 31, 2023. The protocol for online data collection was identical to the laboratory protocol described above. Participants were compensated $20 after the completion of each session.

### 2.6 Automated EEG processing pipeline

An automated processing pipeline was implemented within the MATLAB environment, utilizing a custom script that included the EEGLAB toolbox (Delorme and Makeig, 2004). The same script was applied to each recording.

Files were initially converted to a single EEGLAB data format. A 1–50 Hz bandpass filter was applied to isolate the frequency bands of interest while mitigating the influence of slow drifts and high-frequency noise. A 50- or 60-Hz notch filter was also applied (depending on the geographical location in which each dataset was recorded) to eliminate power-line interference. Files were re-sampled to 125 Hz to reduce the computational demands in downstream analyses. To assure standardization across datasets and correspondence with the test recordings' electrode montage, channels were omitted to match the 16-channel EPOC X configuration (see Figure 1). Data were re-referenced to P3–P4. Channel omission and re-referencing were not applied to the test data. Artifact Subspace Reconstruction (ASR) was then performed utilizing the clean_rawdata plugin (Kothe and Makeig, 2013; Mullen et al., 2015) with the burst criterion parameter set at 5, facilitating the identification and attenuation of artifact-related subspace and enhancing the overall quality of the signal. ASR identifies high-variance segments of data, which are subsequently reconstructed or omitted based on the defined burst criterion. The other functionalities available in the clean_rawdata plugin, including bad-channel rejection and segment rejection were set as “off.”

## 3 Machine learning

### 3.1 Model training

**Figure 2:**
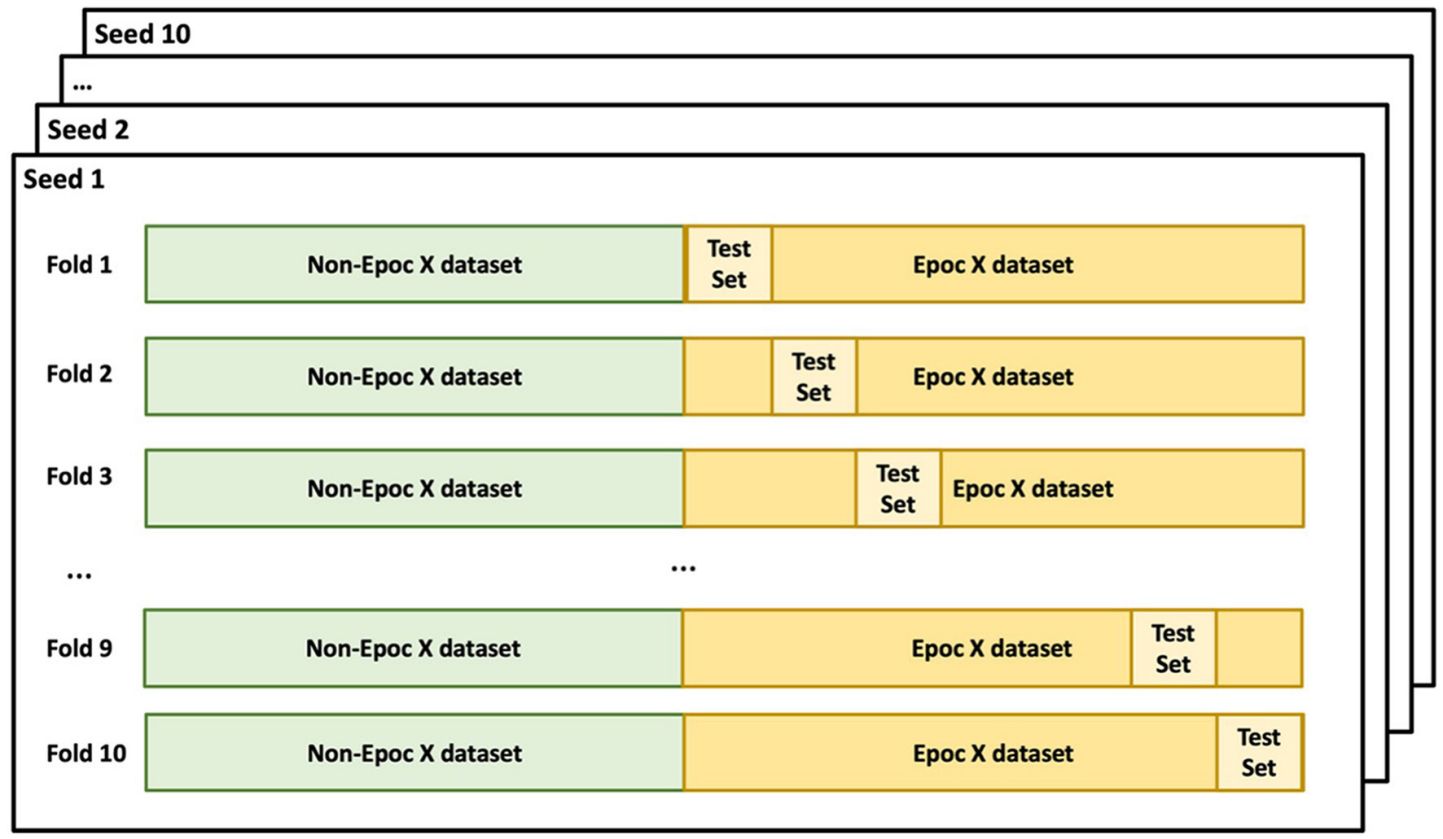
Cross-validation split. Non-EPOC X datasets (supplemental training datasets) were included in the training set for all folds. The EPOC X dataset (SBE dataset) was split into 10-folds based on age. The 10-fold train-test split was repeated 10 iterations using different random seeds.

A machine learning model was developed with the R programming language to predict subjects' chronological ages based on EEG features. The model was trained using a total of 1,215 features including measures of EEG power, connectivity, noise, and variability for nine frequency bands at the 14 electrodes of the EMOTIV EPOC X headset. The dataset was divided based on the recording device: EPOC X (SBE dataset) and non-EPOC X (supplemental training datasets). A stratified 10-fold train-test split based on age was performed for the SBE dataset, ensuring a balanced representation of all age categories in each fold (Figure 2). Conversely, all datasets recorded using non-EPOC X systems were included in the training set. For each fold of the 10-fold split for the SBE dataset, the training set was merged with the non-EPOC X dataset to formulate a comprehensive training dataset. This approach facilitated the incorporation of diverse data while maintaining a structured evaluation strategy for age prediction.

### 3.2 Model development

An ensemble model was trained using three distinct algorithms: [1] Support Vector Regression (SVR) with a radial basis kernel (svmRadial), [2] Elastic net regression (glmnet), and [3] Gaussian Process with Radial Basis Function Kernel (gaussprRadial). This ensemble model aimed to harness the predictive capabilities of each algorithm, ensuring a comprehensive and potentially more accurate brain-age prediction. The model was trained to predict the subject's chronological age using the EEG-derived features extracted in the preceding steps.

### 3.3 Bias correction

Following model training, residuals were computed as the difference between the observed and predicted chronological age for each subject in the training set. A linear model for bias correction was then fitted using these residuals and the actual chronological age, aiming to enhance the prediction accuracy by mitigating any systematic bias in the initial predictions.

As noted in the brain-age prediction literature, brain age is typically underestimated for older subjects and overestimated for younger subjects (Cole et al., 2017; Liang et al., 2019; Smith et al., 2019), and the estimated brain-age gap therefore tends to be negatively correlated with chronological age. Several studies have investigated the possible underlying reasons for this systematic bias and found that it is likely due to regression toward the mean (Liang et al., 2019; Smith et al., 2019; Niu et al., 2020, 2022).

Following the recommendation in the literature, we implemented a general linear model to regress the estimated brain-age gap on chronological age (Beheshti et al., 2019; Liang et al., 2019; Smith et al., 2019; Niu et al., 2020). The linear model for bias correction was fitted using a cross-validation approach using both the training and testing EPOC X datasets to ensure generalizability of the model to the test examples. Each data point of the combined EPOC X data was assigned a binary “Group” variable (training-−0 and testing-−1) based on its origin to facilitate the segregation of training and test sets. Then this merged dataset was two-fold split into training and validation sets while maintaining the ratio of the original training and test examples. During each iteration of the CV, the bias-correction adjustment was modeled as Offset ~ Age^*^Group, and adjusted brain age (Estimated brain age-Offset) and adjusted BAG (adjusted brain age-chronological age) were computed for the validation set.

The estimated parameters were then used for bias correction and the calculation of an adjusted BAG. When evaluating the BAG as a potential biomarker for health outcomes, it is essential to account for such systematic bias by either calculating the adjusted BAG or controlling for chronological age. Otherwise, the effect of the BAG can be confounded with chronological age. It is also important to note that the bias-correction step takes chronological age as an input to the general linear model, making it circular for evaluating model prediction accuracy (Butler et al., 2021). Thus, while bias-corrected brain-age gaps may be used for individual brain-age estimation to avoid underestimating brain ages for older subjects and overestimating brain ages for younger subjects, conservative assessment of model accuracy should rely on the pre-corrected model.

### 3.4 Model evaluation

The test set, comprised exclusively of EPOC X data, was employed to evaluate the model. Mean Absolute Error (MAE), *R*-squared (*R*^2^), and the correlation coefficient, were computed to assess the model's predictive accuracy and goodness of fit. Subsequently, bias correction derived from the training set was applied to the predictions on the test set, and the three measures were recalculated to evaluate the efficacy of the bias correction in enhancing predictive accuracy. The entire 10-fold training and testing procedure was repeated using 10 different seed values, ensuring variability and robustness in the training and evaluation processes.

## 4 Results

The model evaluation performance is reported using correlation coefficient (*r*), *R*^2^, and mean absolute error (MAE). To ensure generalizability and consistency of the results, the train-test split and model training-testing were repeated 10 times using different random seeds. Here, performance was computed based on the average of 10 iterations.

### 4.1 Training-set performance

The correlation coefficient (*r*) between predicted and chronological brain age based on the training set was 0.912 (±0.002 SD, range: 0.909–0.916), suggesting a strong linear relationship. This was affirmed by a coefficient of determination (*R*^2^) of 0.830 (±0.004 SD, range: 0.824–0.837), indicating that 83.0% of the variance in the true brain age was explained by our model's predictions based on the training data. The machine-learning model exhibited a mean absolute error (MAE) of 5.209 years (±0.109 SD, range: 5.051–5.346).

### 4.2 Test-set performance

When assessing the model's performance on the test set without bias correction, the correlation coefficient between the predicted and chronological brain age was 0.582 (±0.002 SD, range: 0.579–0.585), and the *R*^2^ value was 0.292 (±0.002 SD, range: 0.349–0.358). Thus, 35.2% of the variance in the chronological brain age was explained by the model's predictions on the test data. The MAE was 11.08 years (±0.043 SD, range: 10.99–11.14).

### 4.3 Bias-corrected test-set performance

**Figure 3:**
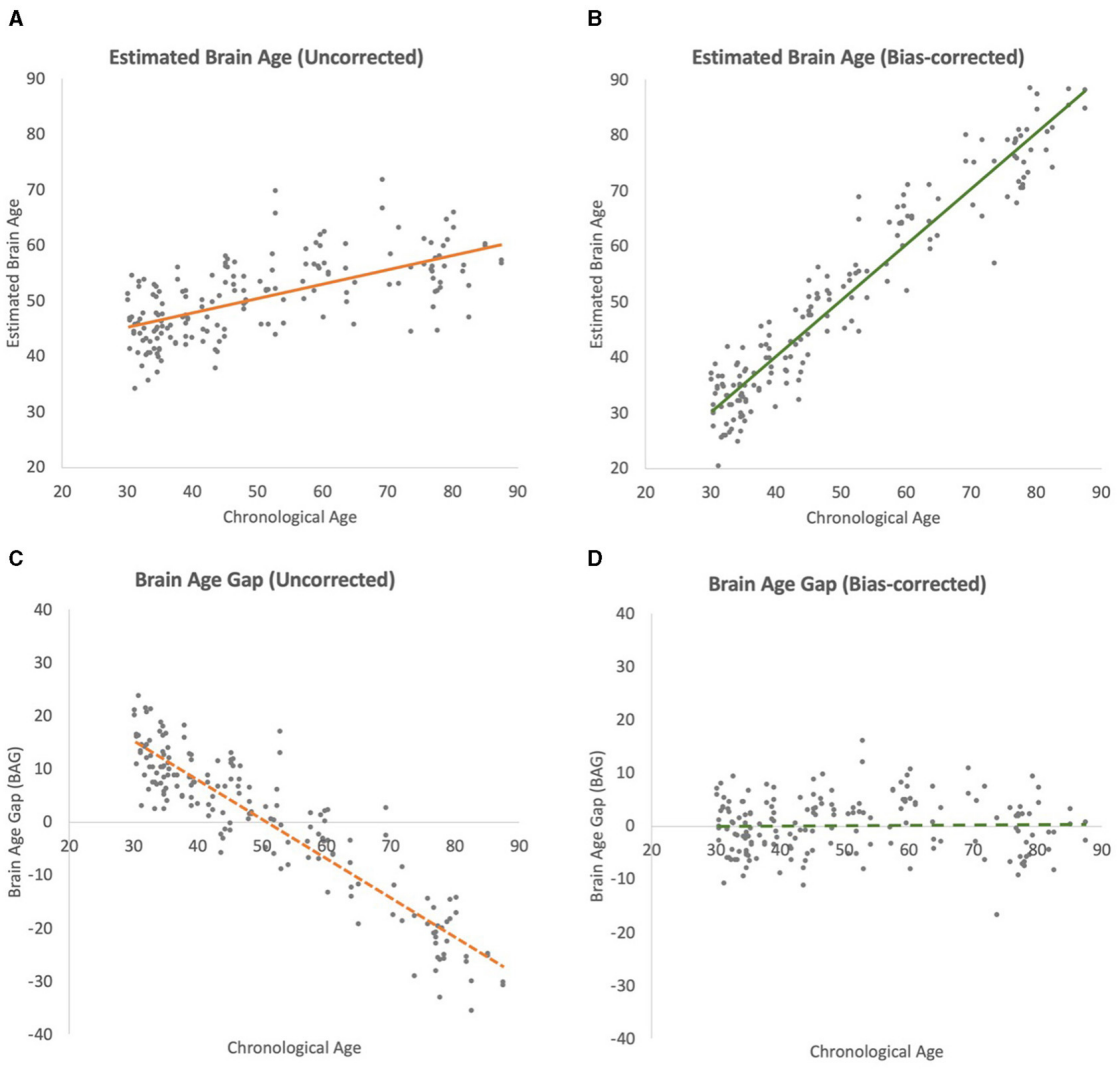
Scatterplots of brain-age estimation outcomes (chronological ages vs. estimated brain ages) of iterations. All regression lines depict linear best fits. **(A)** Uncorrected test-set estimation. **(B)** Bias-corrected test-set estimation. **(C)** Uncorrected brain-age gap (BAG). **(D)** Bias-corrected BAG.

After bias correction was applied to the test-set predictions, there was a substantial improvement in the model's performance (see Figure 3 for results from Seed 1 which is representative of the results from all 10 seeds). The correlation coefficient between the bias-corrected predictions and chronological brain age increased to 0.965 (±0.001 SD, range: 0.964–0.966). The bias-corrected *R*^2^ value was 0.927 (±0.001 SD, range: 0.925–0.929), indicating that the model, after correction, was able to explain ~92.7% of the variance in the true brain age on the test data. The bias-corrected MAE was reduced to 3.681 years (±0.031 SD, range: 3.629–3.729).

### 4.4 Test-retest reliability

**Figure 4:**
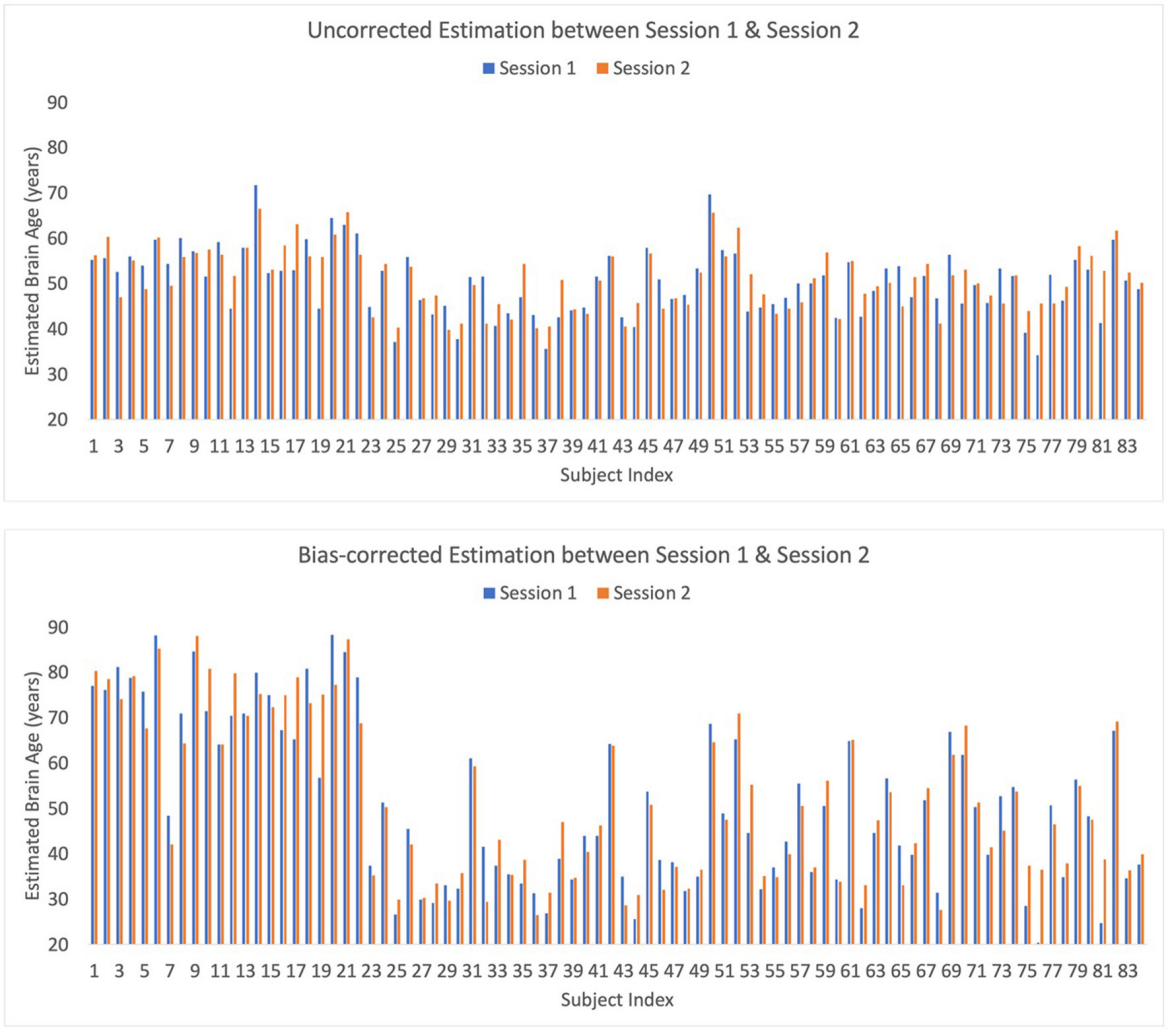
Bar plot of raw and bias-corrected brain ages from Sessions 1 and 2 for Seed 1 which is representative of the results from all 10 seeds.

Eighty-four out of 91 subjects in our SBE dataset participated in two sessions, 1 week apart. This provided a robust dataset that allowed the assessment of test-retest reliability or consistency in brain-age estimations at two close time points. To evaluate this, the correlation coefficient and MAE between the brain-age predictions from Session 1 and Session 2 were calculated. This was achieved by utilizing the left-out test-set from a 10-fold cross-validation procedure and replicating the computation of correlation coefficients and MAEs across 10 different random seeds to ensure the reliability and stability of our results. The average correlation coefficient across these 10 iterations was 0.771 ± 0.003 for raw estimations. When implementing bias-correction, the correlation coefficient was 0.943 ± 0.001, indicating strong test-retest reliability of brain-age prediction. The MAE, which measures the average magnitude of differences between brain-age estimates for Sessions 1 and 2, was calculated to evaluate the absolute accuracy and consistency of the brain-age predictions across the two sessions. The MAE between Session 1 and Session 2 was 3.839 (4.621 after bias correction). Overall, these findings underscore the strong reliability and stability of our brain-age estimates across two separate sessions that were near in time when using the EMOTIV EPOC X recording system (Figure 4).

## 5 Discussion

The current version of our ML-based brain-age estimation technique based on the low-cost EMOTIV EPOC X EEG recording system yields accurate, reliable estimates of a person's brain age from only 12 min of resting-state EEG. Aside from this report, we are aware of only one other study utilizing EEG recordings from a low-cost device for brain-age prediction. Banville et al. (2023) used extended EEG recordings obtained during sleep and meditation, in contrast to our recordings which were obtained during the conscious resting-state. Nevertheless, both studies achieved strong prediction performance suggesting the feasibility of using low-cost EEG devices for monitoring general brain health and aging. However, in contrast to Banville et al., our study achieved high short-term test-retest reliability and stability, suggesting the potential of our approach for reliably assessing changes in brain health via repeated testing. However, the observed strong test-retest reliability was primarily centered on 2-week time span which could be considered short-term, and it was due to the constraints in the study period and estimates. We acknowledge that it is critical to evaluate the stability of the model over extended period of time, and future research should be directed toward longitudinal studies which would provide a deeper insight into the temporal stability of our brain-age estimation model. In addition, future research is needed to validate our findings using a larger and more diverse sample. Dimension reduction techniques such as recursive feature elimination and principal component analysis hold the potential to further increase the accuracy of brain age prediction. Future studies can explore additional EEG features to further improve the model prediction performance.

Our approach to EEG brain-age estimation has several promising applications. It can be used as a relatively inexpensive screening tool to identify individuals whose brain-age gap suggests the possibility of underlying age-related pathology that can be followed up with specific diagnostic tests. Furthermore, because of the relatively low cost of the EMOTIV EPOC X headset, EEG brain-age estimation can be performed repeatedly to verify results and detect changes over time. This means that it may become practical to begin screening people in early middle age (or younger) rather than waiting for late middle age, or older, when symptoms become apparent; implementation of the model for large-scale assessment will require the enactment of appropriate ethical and privacy protections. This raises the possibility of large-scale detection and treatment of the earliest phases of age-related neurological disorders rather than waiting for the overt symptomatology characteristic of advanced—and currently untreatable—pathology. It may also be a useful tool for researchers, medical professionals, and others who wish to test potential interventions for slowing or reversing neurological aging. The current availability of inexpensive EEG systems such as the EPOC X also makes brain-age estimation possible at home or in the workplace, thereby raising the possibility of crowd-sourced research into causes and potential treatments for age-related neurocognitive decline and lifestyle interventions that may preserve neurocognitive health.

## Data availability statement

The study used five combined datasets. Two are publicly available (Cuban Human Brain Mapping Project and the Stimulus-Selective Response Modulation Project). These datasets are cited in the article and are of sufficient size to enable anyone to check our basic findings. The three other datasets (Stockton A, Stockton B, and Test Set) are proprietary to Stockton and Drexel Universities and are not publicly available.

## Ethics statement

The studies involving humans were approved by Drexel University, Stockton University, Bryn Mawr College. The studies were conducted in accordance with the local legislation and institutional requirements. The participants provided their written informed consent to participate in this study.

## Author contributions

JK: Conceptualization, Funding acquisition, Investigation, Methodology, Project administration, Supervision, Writing—original draft, Writing—review & editing. JF: Conceptualization, Funding acquisition, Investigation, Methodology, Project administration, Supervision, Writing—original draft, Writing—review & editing. FZ: Conceptualization, Formal analysis, Investigation, Methodology, Software, Supervision, Writing—original draft, Writing—review & editing. YO: Conceptualization, Investigation, Methodology, Software, Writing—original draft, Writing—review & editing.
